# Association between dietary magnesium intake and pelvic inflammatory disease in US women: a cross-sectional study of NHANES

**DOI:** 10.3389/fnut.2024.1430730

**Published:** 2024-08-07

**Authors:** Zeru Chen, Zichun Wu, Yuying Zhang

**Affiliations:** ^1^Department of Clinical Medicine, The Second School of Clinical Medicine, Guangzhou Medical University, Guangzhou, China; ^2^Department of Clinical Medicine, The First School of Clinical Medicine, Guangzhou Medical University, Guangzhou, China; ^3^Department of Gynecology, Shenzhen Longhua Maternity and Child Healthcare Hospital, Shenzhen, China

**Keywords:** magnesium, pelvic inflammatory disease, National Health and Nutrition Examination Survey, cross-sectional study, restricted cubic spline

## Abstract

**Background:**

Pelvic inflammatory disease (PID) is a common gynecological condition associated with significant morbidity and healthcare costs. Emerging evidence suggests that dietary factors, such as magnesium intake, may play a role in PID risk. However, the relationship between dietary magnesium intake and PID risk remains uncertain. This cross-sectional study aimed to investigate the association between dietary magnesium intake and the risk of PID.

**Methods:**

This cross-sectional study included data from the National Health and Nutrition Examination Survey (NHANES) 2015–2018. Weighted multivariable logistic regression was used to examine the association between dietary magnesium intake and PID. Restricted cubic spline (RCS) analysis was performed to assess the linear and non-linear associations. Subgroup analyses were performed based on baseline characteristics.

**Results:**

A total of 3,034 women aged 20–59 were included in the study. Magnesium intake exhibited a significant association with lower PID risk in weighted multivariable logistic regression. Adjusted odds ratios (ORs) for dietary magnesium intake in quartiles Q2 (133.12–214.93 mg/day), Q3 (214.93–287.19 mg/day), and Q4 (above 287.19 mg/day) compared to Q1 (below 133.12 mg/day) were 0.48 (95% CI: 0.28–0.82), 0.64 (95% CI: 0.32–1.27), and 0.40 (95% CI: 0.18–0.88), respectively. Stratified analyses showed that significant association between dietary magnesium intake and PID in older subgroup but not in younger subgroup. Additionally, RCS analyses consistently revealed a linear negative correlation between dietary magnesium intake and PID risk.

**Conclusion:**

This study reveals a significant negative correlation between dietary magnesium intake and risk of PID, particularly among older individuals. These findings underscore the importance of dietary factors in gynecological health and highlight the potential role of magnesium supplementation in PID prevention strategies.

## 1 Introduction

Pelvic Inflammatory Disease (PID) is a commonly encountered inflammatory ailment, primarily affecting sexually active young females. It is characterized by infections in the upper reproductive system, specifically targeting the uterus, fallopian tubes, ovaries, and pelvic peritoneum (1). Without prompt treatment, PID harbors the potential to culminate in dire consequences, including ectopic pregnancy, infertility, persistent pelvic pain, and adverse pregnancy outcomes (2). Despite its high incidence, PID can be prevented and managed through lifestyle modifications, including dietary changes (3, 4).

Magnesium, a vital element abundantly present in daily meals, is crucial for DNA and RNA synthesis, cell growth, and protein production, ranking as the human body’s fourth most important mineral (5, 6). Research indicates that the dietary magnesium intake in developed countries has declined over recent decades due to the increased consumption of processed and fast foods (7). This trend has particularly impacted the United States, where most adults, especially young girls and women, fall short of reaching the recommended daily intake of magnesium (8–10). Insufficient magnesium intake is associated with a series of chronic conditions, including cardiovascular diseases, type 2 diabetes mellitus, osteoporosis, lung diseases, depression, migraines, and inflammatory diseases (11). Intriguingly, an inverse correlation exists between the intake of dietary magnesium and the levels of serum C-reactive protein (CRP) (12–14). However, the connection between the consumption of dietary magnesium and the incidence of PID remains to be established.

In this study, we analyzed data from the National Health and Nutrition Examination Survey (NHANES) (2015–2018) to uncover the relationship between the intake of dietary magnesium and the likelihood of developing PID. Our study may provide new insights for the prevention and clinical management of PID.

## 2 Materials and methods

### 2.1 Data source and participants

This study utilized a cross-sectional research approach, utilizing data from the NHANES, a comprehensive, multistage, stratified, and complex survey conducted nationally by the Centers for Disease Control and Prevention (CDC). Participants underwent initial interviews in their homes, followed by a series of clinical and laboratory tests at Mobile Examination Centers (MEC). Detailed information about the survey and associated research data is available on the NHANES website at https://www.cdc.gov/nchs/nhanes/.

**Figure 1:**
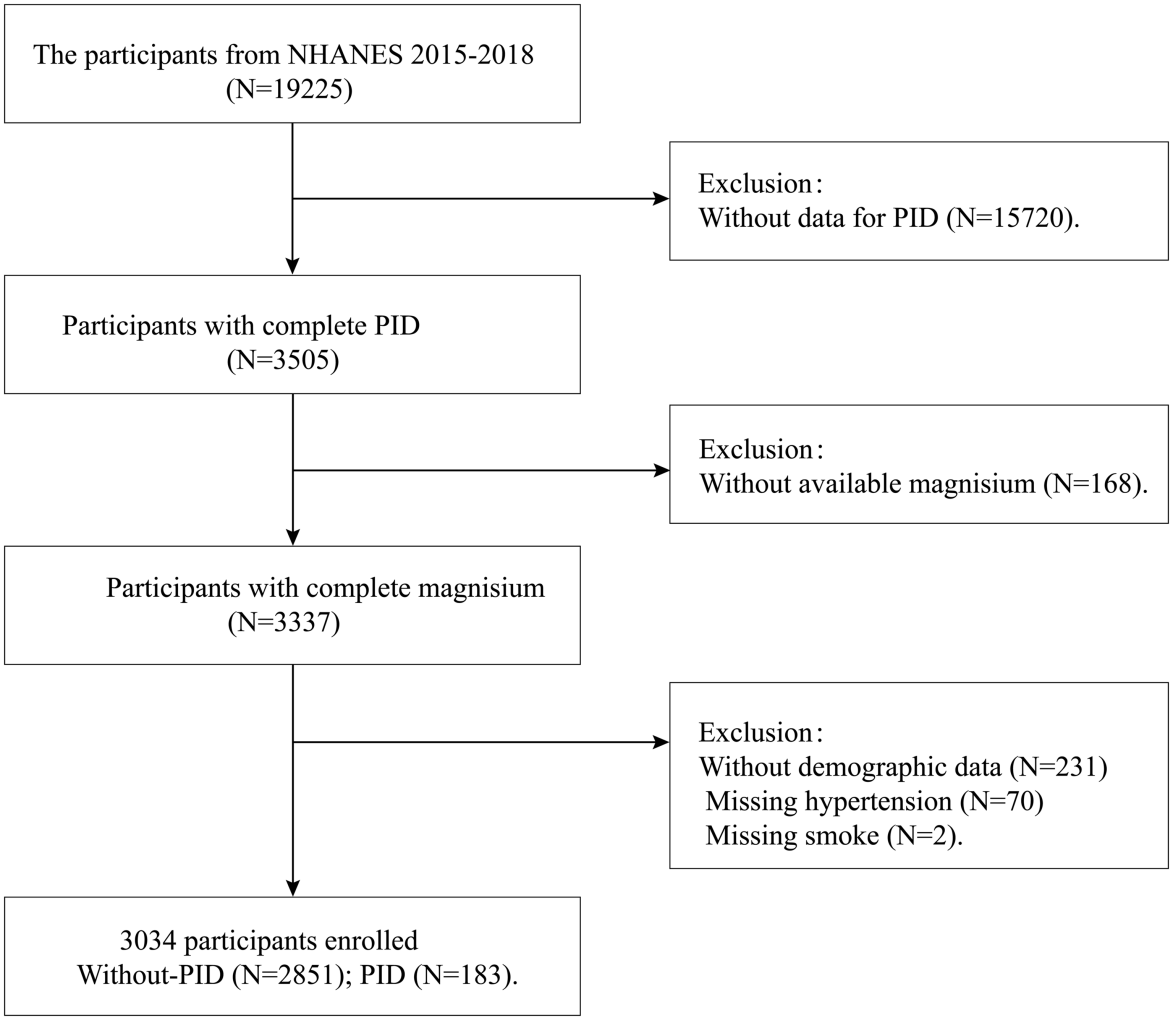
Flowchart of participant selection from NHANES 2015–2018.

The study selected participant data from two NHANES survey cycles (2015–2016 and 2017–2018). Initially, 19,225 female participants were included. In the second step, 15,720 samples lacking PID data were excluded. In the third step, 168 samples with missing dietary magnesium data were excluded. Additionally, 303 participants with missing demographic information, hypertension data, and smoking data were considered ineligible. Ultimately, a total of 3,034 women aged 20–59 were included in our final analysis, of which 183 had PID. Additionally, our study employed weighted adjustments to accurately reflect the demographic composition of the United States population. The flowchart detailing the inclusion and exclusion criteria is shown in Figure 1.

The NHANES initiative received ethical clearance from the Ethical Review Committee of the National Center for Health Statistics, with all participants providing written informed consent. Our study rigorously adhered to the protocols outlined by the Strengthening the Reporting of Observational Studies in Epidemiology (STROBE) criteria (15).

### 2.2 Assessment of dietary magnesium intake

The NHANES study utilized a 24-h dietary recall questionnaire, administered to all study participants. This questionnaire collected comprehensive information on all foods and beverages consumed in the past 24 h, detailing the types and quantities of each item (16). For each NHANES participant from the 2015–2018 cycles, two 24-h recalls were conducted. The first dietary recall was performed at the NHANES Mobile Examination Center (MEC), and the second recall was conducted by trained interviewers via telephone 3–10 days after the MEC interview. We calculated the average dietary magnesium intake from the two recall periods; if the second recall was unavailable, the data from the first recall was used. To accurately estimate the dietary magnesium consumption, the Food Intake Analysis System developed by the University of Texas was used, in conjunction with the USDA’s National Nutrient Database for Standard Reference.

### 2.3 Assessment of pelvic inflammatory disease

The diagnosis of PID was assessed using data obtained from the NHANES Reproductive Health questionnaire. Specifically, question RHQ078 asked: “Have you ever been treated for an infection in your fallopian tubes, uterus, or ovaries, also called a pelvic infection, pelvic inflammatory disease, or PID?” Participants who answered “Yes” to this question were considered to have PID.

### 2.4 Covariates

Consistent with previous pertinent research (4, 16–18), demographic factors (age, race, educational background, marital status), socioeconomic status [Poverty Income Ratio (PIR)], health and lifestyle indicators [Body Mass Index (BMI), smoke status, presence of chronic conditions such as diabetes and hypertension], and reproductive health metrics (regularity of menstrual periods) were included as covariates in our analysis. Age was classified into two groups: 20–40 years and 41–59 years. Race was classified into four categories: Non-Hispanic White, Mexican American, Non-Hispanic Black, and other races. Meanwhile, educational background was organized into three tiers: below high school, high school, and above high school (19). Marital status was categorized as married or living with a partner or living alone (20). NHANES meticulously recorded participants’ smoking behaviors, including status, duration, and associated activities (21). Smoking status was categorized as follows (22): never smokers are adults who have not smoked or have smoked less than 100 cigarettes in total; former smokers have smoked 100 or more cigarettes but no longer smoke; current smokers have smoked over 100 cigarettes and continue to smoke, whether occasionally or daily. For the evaluation of diabetes, participants were asked, “Have you ever been told by a doctor or health professional that you have diabetes or sugar diabetes?” Respondents affirming this question were classified as diabetic (23). Hypertension was diagnosed by averaging three measurements of resting brachial arterial pressure, with the criteria being a systolic pressure of 140 mmHg or higher and a diastolic pressure of 90 mmHg or higher (24). The PIR compares household income to the poverty line, indicating socioeconomic status. Categories include low (PIR under 1.35), middle (PIR 1.35–3.0), and high (PIR over 3.0) (25, 26). BMI is classified into normal weight (below 25.0 kg/m^2^), overweight (25.0–29.9 kg/m^2^), and obesity (above 30.0 kg/m^2^) (27). Regular period data were assessed through the reproductive health questionnaire that asked, “Had regular periods in past 12 months (eligible for participants aged 12 years older)?,” and women who were pregnant or who had bleeding as result of medical issues, hormonal treatments, or surgical procedures were excluded.

### 2.5 Statistical analysis

All statistical analyses in this study were conducted using dietary weights, ensuring precision and integrity in line with the least common denominator strategy guided by NHANES sampling weights (28). Continuous data were presented as mean values with standard deviations (SD), while categorical data were presented as counts (n) and proportions (%). To compare the baseline characteristics between the PID and non-PID groups, we employed the Mann–Whitney U test or *t*-test for continuous variables, and the chi-square test or Fisher’s exact test for categorical variables.

To elucidate the relationship between dietary magnesium intake and PID, weighted multivariable logistic regression analyses were conducted. Initially, magnesium intake was evaluated as a continuous variable and then divided into quartiles for detailed analysis: Q1 (below 133.12 mg/day), Q2 (133.12–214.93 mg/day), Q3 (214.93–287.19 mg/day), and Q4 (above 287.19 mg/day), with Q1 serving as the baseline comparison group. We tested different models (Models 1–3) by progressively adjusting for various risk factors. Model 1 remained unadjusted, Model 2 adjusted for key confounders including age and race, and Model 3 further adjusted for education level, marital status, PIR, BMI, regularity of menstrual periods, smoking status, and presence of chronic conditions including diabetes and hypertension. After converting dietary magnesium intake from a continuous to a categorical variable, trend tests were conducted to examine the linear relationship trend between dietary magnesium consumption and PID. Restricted cubic splines (RCS) were utilized to evaluate the nonlinear and dose–response relationships between the intake of dietary magnesium and PID. Additionally, stratified analyses and the inclusion of interaction terms were employed to examine the consistency of this association across different demographic groups. Multiple imputation (MI) is a method for addressing missing data by creating multiple datasets (29). The MI program uses the chained equations method to impute missing values for various variables, including education, PIR, BMI, and diabetes.

The association between magnesium intake and PID is expressed through odds ratios (ORs). Analyses were performed with R (version 4.2) and EmpowerStats (version 5.0). A two-sided *p*-value below 0.05 was considered statistically significant.

## 3 Results

### 3.1 Baseline characteristics of the participants

**Figure 2:**
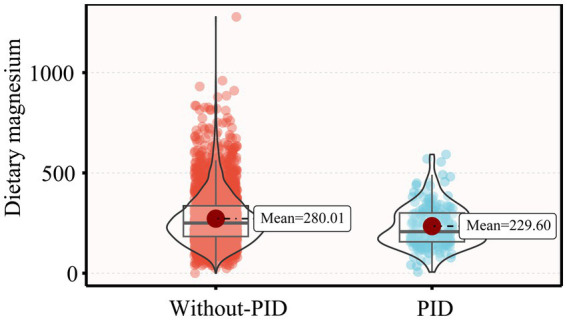
Dietary magnesium intake levels in participants with or without PID.

Table 1 presents the weighted demographic and baseline characteristics of the study participants. This study included 3,034 participants, with an average age of 40.01 years. The majority were Non-Hispanic White, with a high school education or higher, and either married or living with a partner. Compared to participants without PID, those diagnosed with PID were older, more likely to smoke and experience irregular menstrual cycles, had lower family income, higher BMI, and lower dietary magnesium intake levels (229.60 mg/day, *p* < 0.001) (Figure 2).

**Table 1:** Characteristics of the women participants, weighted.

Variables	Overall (*n* = 3,034)	Without PID (*n* = 2,851)	PID (*n* = 183)	*p*-value
Magnesium (mg/day)	277.11 (127.07)	280.01 (127.77)	229.60 (104.33)	<0.001
Age (years)	40.01 (11.87)	39.82 (11.89)	43.08 (11.00)	0.007
**Race (*n*, %)**				0.273
Non-Hispanic White	1,776 (58.55)	1,678 (58.87)	98 (53.33)	
Non-Hispanic Black	385 (12.68)	353 (12.38)	32 (17.49)	
Mexican American	312 (10.28)	300 (10.51)	12 (6.60)	
Other races	561 (18.50)	520 (18.24)	41 (22.58)	
**Education (*n*, %)**				0.134
Below high school	309 (10.20)	283 (9.91)	27 (14.92)	
High school	674 (22.20)	620 (21.76)	54 (29.43)	
Above high school	2,051 (67.60)	1,948 (68.33)	102 (55.65)	
**Marital (*n*, %)**				0.184
Living alone	1,139 (37.53)	1,058 (37.12)	81 (44.36)	
Living with a partner	1,895 (62.47)	1,793 (62.88)	102 (55.65)	
**PIR (*n*, %)**				0.002
Low-income family	749 (24.68)	676 (23.71)	74 (40.50)	
Middle-income family	1,069 (35.23)	996 (34.94)	73 (40.10)	
High-income family	1,216 (40.09)	1,179 (41.35)	36 (19.40)	
**BMI (*n*, %)**				0.004
Normal weight	978 (32.22)	952 (33.38)	24 (13.22)	
Overweight	783 (25.80)	720 (25.25)	64 (34.77)	
Obese	1,273 (41.98)	1,179 (41.37)	95 (52.01)	
**Smoke (*n*, %)**				<0.001
Never	1,980 (65.25)	1,908 (66.93)	69 (37.66)	
Former	473 (15.60)	429 (15.06)	45 (24.43)	
Now	581 (19.15)	514 (18.01)	69 (37.91)	
**Hypertension (*n*, %)**	273 (9.00)	251 (8.80)	23 (12.34)	0.135
**Diabetes mellitus (*n*, %)**				0.298
Pre-diabetes mellitus	56 (1.83)	54 (1.91)	1 (0.55)	
Diabetes mellitus	201 (6.62)	189 (6.63)	12 (6.60)	
**Regular period (*n*, %)**	1,975 (65.10)	1,878 (65.88)	96 (52.37)	0.003

For continuous variables: survey-weighted mean (SD), *p*-value was by survey-weighted linear regression (svyglm); for categorical variables: survey-weighted *n* (%), *p*-value was by survey-weighted Chi-square test (svytable).

PID, Pelvic Inflammatory Disease; PIR, Poverty Income Ratio; BMI, Body Mass Index.

*p*-value less than 0.05 is expressed in bold.

### 3.2 The associations between dietary magnesium intake and PID

Table 2 illustrates the association between dietary magnesium intake and the risk of developing PID. When dietary magnesium was treated as a continuous variable, a significant correlation was observed across all three models (Models I–III). Specifically, for each 1 mg/day increment in dietary magnesium intake, there was a notable reduction in PID risk, with odds ratios (OR) of 0.996 (0.994–0.998), 0.996 (0.994–0.999), and 0.997 (0.995–1.000) in Models I, II, and III, respectively. These findings suggest a robust relationship between higher dietary magnesium consumption and a reduced incidence of PID.

**Table 2:** Weighted multivariable logistic regression to assess the association of magnesium intake with PID.

	Model I		Model II		Model III	
	OR (95% CI)	*p*-value	OR (95% CI)	*p*-value	OR (95% CI)	*p*-value
Magnesium	0.996 (0.994–0.998)	0.002	0.996 (0.994–0.999)	0.004	0.997 (0.995–1.000)	0.024
Categories						
Q1	1.0 [Ref]		1.0 [Ref]		1.0 [Ref]	
Q2	0.430 (0.265–0.699)	0.001	0.438 (0.264–0.727)	0.003	0.480 (0.281–0.821)	0.012
Q3	0.512 (0.294–0.889)	0.019	0.528 (0.294–0.948)	0.034	0.639 (0.323–1.265)	0.175
Q4	0.294 (0.147–0.587)	0.001	0.299 (0.144–0.619)	0.002	0.395 (0.177–0.879)	0.027
*P* for trend	0.691 (0.559–0.854)	0.002	0.697 (0.559–0.868)	0.004	0.766 (0.605–0.968)	0.046

Magnesium enter as a continuous variable per 1 mg/day increase.

Model I: no covariates were adjusted.

Model II: Adjusted for age, race.

Mode III: Adjusted for age, race, education, marital status, PIR, BMI, regular period, smoke, hypertension, and diabetes.

CI, confidence interval; OR, odds ratios; Ref, reference.

Magnesium is in the quartile: Q1 (<133.12 mg/day), Q2 (133.12–214.93 mg/day), Q3 (214.93–287.19 mg/day), Q4 (>287.19 mg/day).

Additionally, when categorizing dietary magnesium intake into quartiles, the fully adjusted model (Model III) revealed that compared to individuals in the lowest quartile Q1 (less than 133.12 mg/day), the adjusted ORs for participants in Q2 (133.12–214.93 mg/day), Q3 (214.93–287.19 mg/day), and Q4 (more than 287.19 mg/day) were 0.480 (95% CI: 0.281–0.821, *p* = 0.012), 0.639 (95% CI: 0.323–1.265, *p* = 0.175), and 0.395 (95% CI: 0.177–0.879, *p* = 0.027), respectively (Table 2). Trend analysis showed that the likelihood of PID decreased by 23.4% with each quartile increase in dietary magnesium intake (adjusted OR = 0.766, 95%CI: 0.605–0.968, *p* = 0.046).

### 3.3 Restricted cubic spline curve fitting

**Figure 3:**
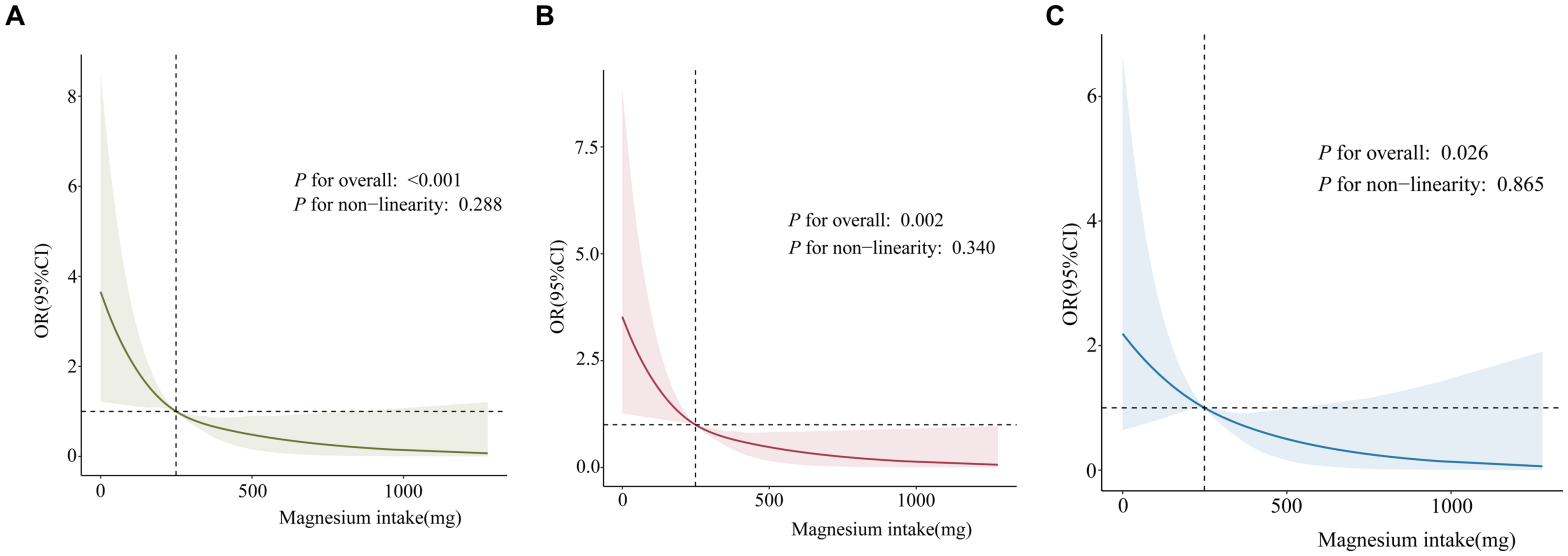
RCS curve fit between dietary magnesium intake and PID. Solid lines represent smooth curve fits between variables. Shaded bands represent 95% confidence intervals from the fit. **(A)** No adjustment for covariates. **(B)** Adjusted for age, and race. **(C)** Adjusted for age, race, education level, marital status, poverty income ratio, body mass index, hypertension, diabetes, smoke and regular period. PID, Pelvic Inflammatory Disease; OR, odds ratio; CI, confidence interval.

Restricted cubic spline (RCS) plots were employed to visualize the correlation between dietary magnesium intake and PID. Figure 3A elucidates the non-adjusted dose–response curve between dietary magnesium intake and PID, while Figures 3B,C show the RCS curve adjusted for covariates, similar to Model II and Model III, respectively. These findings demonstrate a gradual decline in the incidence of PID with higher dietary magnesium intake. Overall, there is a linear negative relationship between dietary magnesium intake and PID, with the relationship being particularly pronounced in Subfigure C (*P* for non-linearity = 0.865).

### 3.4 Subgroup analyses

**Figure 4:**
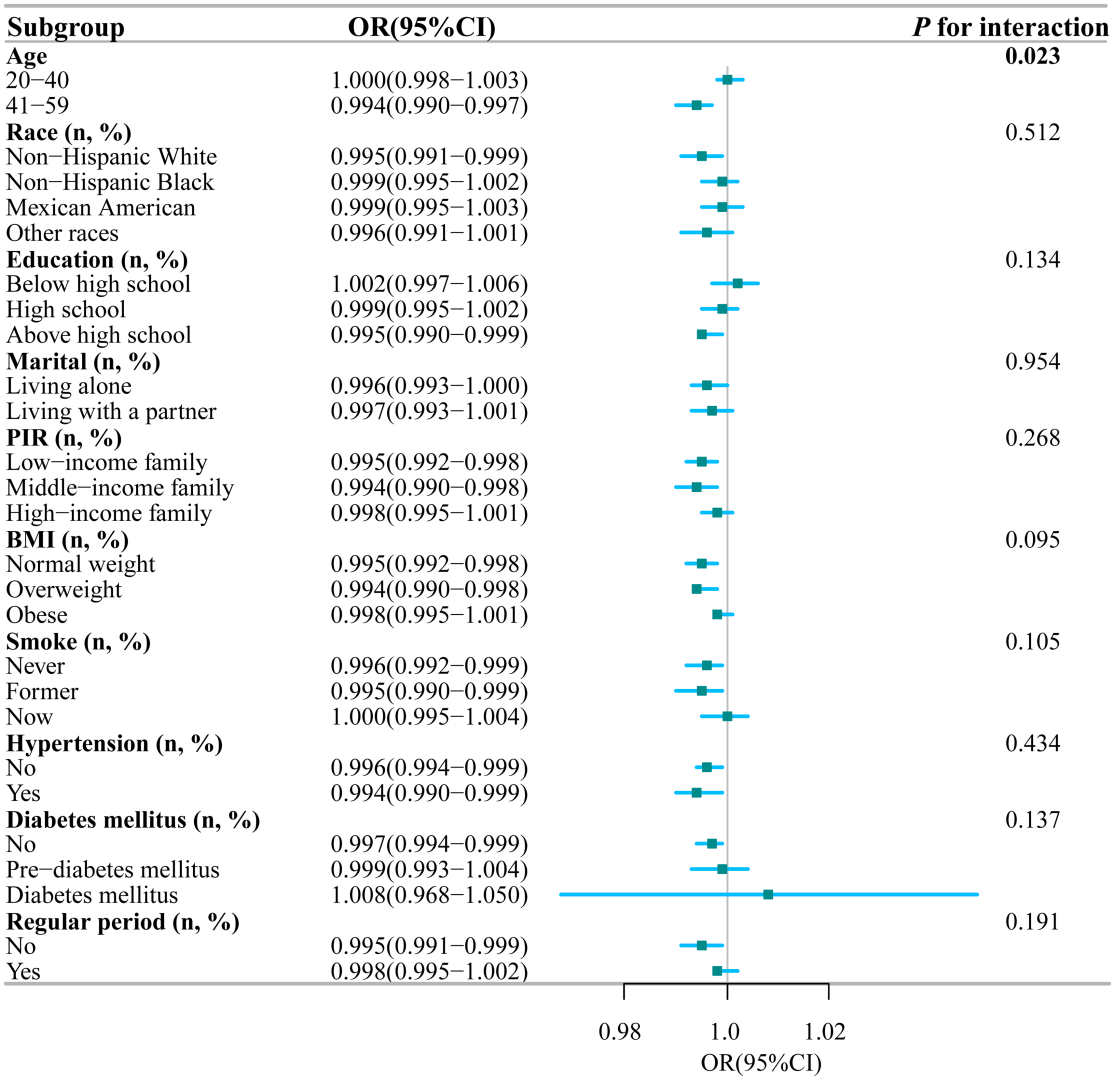
Stratified analyses of the association between dietary magnesium intake and PID according to baseline characteristics. The *p* value for interaction represents the likelihood of interaction be-tween the variable and PID. PIR, Poverty Income Ratio; BMI, Body Mass Index; PID, Pelvic Inflammatory Disease; OR, odds ratio; CI, confidence interval.

**Figure 5:**
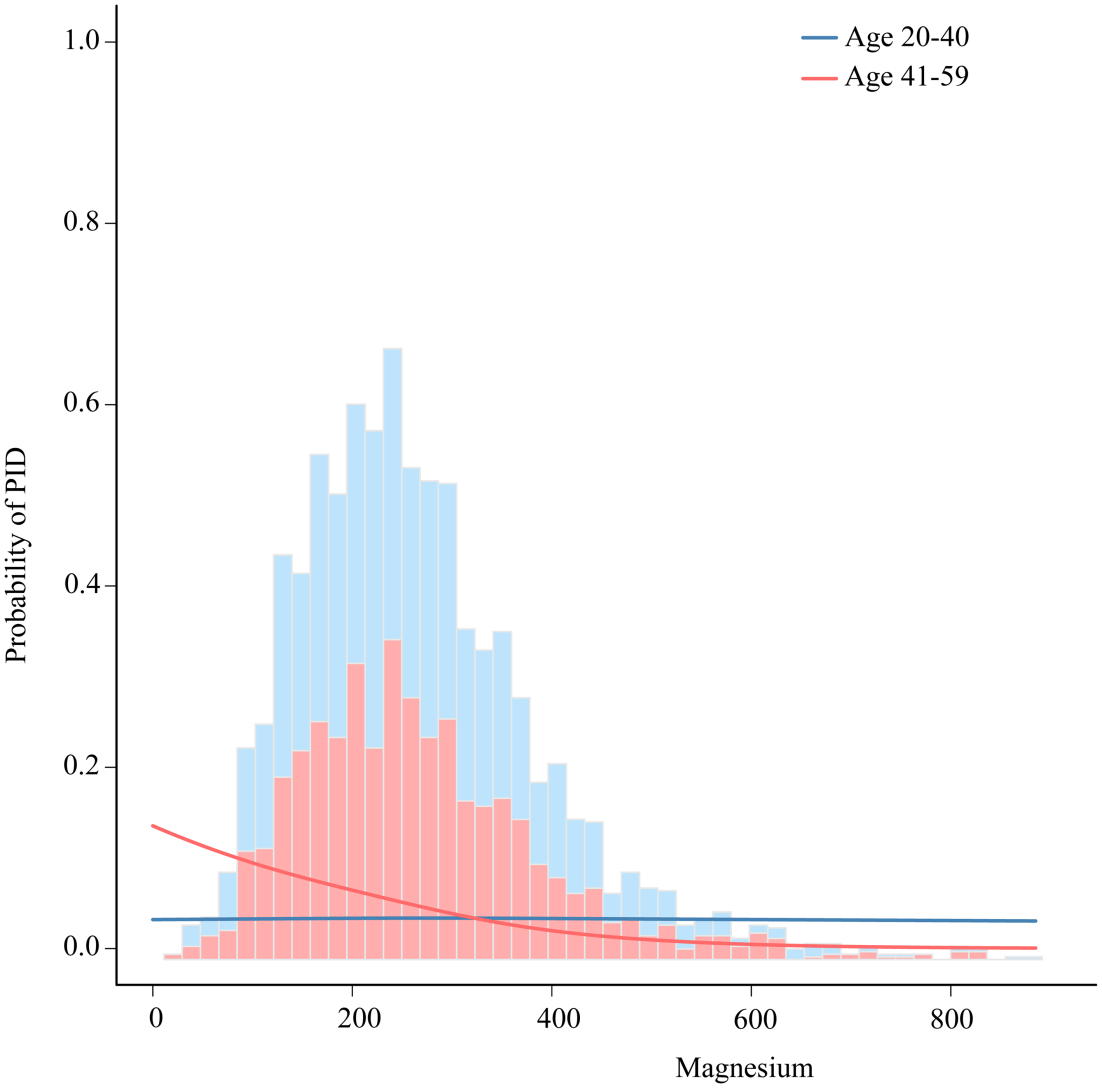
The association between dietary magnesium intake and PID stratified by age. Each stratification adjusted for all the factors (race, education, marital status, body mass index, poverty income ratio, smoke, diabetes, hypertension and regular period). PID, Pelvic Inflammatory Disease; OR, odds ratio; CI, confidence interval.

To further explore the potential link between the intake of dietary magnesium and PID, stratified analysis was performed for various subgroups within Model III (Figure 4). The analysis revealed a significant negative correlation between dietary magnesium consumption and PID among several subgroups. Specifically, elderly women, individuals with lower to middle household incomes, those with normal weight or overweight, non-smokers, individuals without diabetes, and those experiencing irregular menstrual cycles all showed a notable inverse relationship between dietary magnesium intake and PID (all *p*-values < 0.05). Of particular interest, the analysis identified a statistically significant interaction effect with age (interaction *p*-value = 0.023), indicating that the impact of age on this association is nuanced and warrants further investigation. To elucidate this interaction, we depicted the age-dependent relationship between dietary magnesium intake and PID using stratified smoothing curves in Figure 5. These curves provide a visual representation of how the relationship between dietary magnesium intake and PID varies across different age groups, offering deeper insights into the underlying dynamics of this association.

## 4 Discussion

To our knowledge, this is the first study to utilize multivariate logistic regression analysis to investigate the relationship between dietary magnesium intake and the risk of developing PID. In this cross-sectional study with 3,034 participants, we found that higher dietary magnesium intake significantly reduced the likelihood of developing PID. Specifically, compared to the lowest quartile (Q1), participants in the highest quartile (Q4) had a 60.5% lower risk of PID (*p* = 0.027). This negative correlation remained significant even after adjusting for potential confounding variables. Subgroup analysis revealed a significant interaction effect of age on the relationship between dietary magnesium intake and PID. These findings suggest that increasing dietary magnesium intake may be a promising strategy for reducing the incidence of PID.

Previous research have explored the associations between various vitamins and minerals and PID. For example, a previous study in Zimbabwe demonstrated that a single dose of 400,000 IU vitamin A administration at less than 96 h postpartum significantly reduced the risk of late-term reproductive tract infections, consequently decreasing the number of PID-related medical visits among HIV-positive women (30). Besides, Çekmez and his colleagues found that women with PID exhibited considerably higher levels of serum Vitamin D than their non-PID counterparts (31). Additionally, Hu’s study found a significant correlation between reduced dietary copper intake and a lower incidence of PID (4). Our study uniquely highlights a negative association between dietary magnesium intake and PID risk, providing a novel perspective and detailed analysis not clearly established in prior research.

Recent human studies have indicated that magnesium supplementation can significantly reduce various inflammatory markers, particularly C-reactive protein (CRP) (32, 33). Lower CRP levels have been associated with effective management and control of PID (34). These findings suggest that increasing dietary magnesium intake may play a preventative role against PID infections. Furthermore, magnesium supplements have been shown to effectively alleviate pelvic pain symptoms (35, 36), underscoring their therapeutic potential in the treatment of PID. Given the existing evidence, our findings are biologically plausible and introduce a new dimension to the understanding of dietary magnesium’s role in PID prevention and management.

The precise mechanism linking dietary magnesium intake and PID remains incompletely elucidated, with potential mechanisms including immune modulation and reduction of oxidative stress. Studies have indicated that magnesium exhibits inhibitory effects on inflammatory responses and interacts with the immune system, thereby enhancing both innate and adaptive immunity (37). PID commonly arises from microbial infections such as *Chlamydia trachomatis* and *Neisseria gonorrhoeae*, and the subsequent immune responses (38). Given the pivotal role of magnesium in sustaining a robust immune system, increasing dietary magnesium intake could bolster immune responses, potentially reducing the risk of developing PID. Furthermore, oxidative stress is considered a contributing factor to chronic PID (39). PID diminishes the activity of superoxide dismutase (SOD) while increasing malondialdehyde (MDA) levels (40). Interestingly, increased dietary magnesium intake has been demonstrated to lower MDA levels, effectively mitigating oxidative stress (41, 42). Conversely, inadequate magnesium intake has been identified as a cause of oxidative stress. Therefore, enhancing dietary magnesium intake may mitigate oxidative stress by reducing MDA levels, consequently lowering the risk of pelvic inflammatory disease.

In this study, the effect of dietary magnesium intake on the risk of PID varied by age. Stratified analysis revealed a significant inverse association between dietary magnesium intake and PID in older adults, whereas no such association was observed in younger adults. Our finding aligns with a prior study that demonstrated a significant inverse relationship between dietary magnesium intake and anemia, particularly among the elderly population (age > 60 years) (43). Several factors may explain this age-related difference. Biologically, older adults may have a diminished ability to absorb and retain magnesium due to age-related changes in digestive and kidney functions. As individuals age, their digestive efficiency declines, impairing magnesium absorption (44), while the kidneys’ capacity to regulate magnesium weakens, resulting in increased excretion (45). Additionally, older adults may have different dietary patterns and lifestyle factors compared to younger individuals, which can influence magnesium intake and absorption. For example, older adults are more likely to consume magnesium-rich foods or supplements due to increased health awareness or medical advice (46). Furthermore, the prevalence of chronic conditions that benefit from magnesium intake is higher among older individuals, making the effects of dietary magnesium more pronounced in this age group (44). Therefore, ensuring an adequate supply of magnesium is particularly important for older adults.

Our findings hold significant implications for clinical practice, research, and health policymaking. To our knowledge, antibiotics are the mainstay of PID treatment (47, 48). However, the surge in antibiotic resistance underscores the urgent need for alternative preventive and treatment strategies. Our study suggests that increased dietary magnesium intake could serve as a non-pharmacological prevention method. For at-risk women, it is recommended to meet the daily magnesium intake of 300 mg, as advised by the NIH. This can be achieved by consuming magnesium-rich foods such as a handful of dry roasted almonds (80 mg), half a cup of cooked spinach (78 mg), and half a cup of black beans (60 mg). Ensuring adequate magnesium intake may help lower PID risk.

The study has several strengths. Based on the comprehensive NHANES database, encompassing a vast population sample and incorporating appropriate NHANES sample weights, our analysis enhances the representativeness of our results. Additionally, rigorous adjustment for confounding covariates in our model ensures the reliability and wider applicability of our findings. However, several limitations of this study deserve attention. Firstly, due to its cross-sectional design, the causal effect of magnesium on PID cannot be determined. Secondly, questionnaires and other measures introduce a latent information bias in assessing dietary magnesium intake and PID prevalence. Thirdly, data from NHANES is the only source of the study, which may restrict the study’s generalizability. Further research is needed to establish the causal relationship between dietary magnesium intake and PID, as well as to investigate optimal intake levels of magnesium, considering individual variability and dietary patterns.

## 5 Conclusion

This study provides evidence that higher dietary magnesium intake may be protective against PID, particularly in older individuals. These findings highlight the significant role of dietary factors in gynecological health and suggest that magnesium supplementation may be a valuable strategy in PID prevention. However, due to the cross-sectional design and the presence of potential confounding factors, these results should be interpreted with caution. Further longitudinal and interventional studies are needed to confirm causality and explore biological mechanisms. It is also crucial to delve into the optimal levels of magnesium intake and how they interact with other dietary components and lifestyle factors, in order to formulate optimal prevention strategies.

## Data availability statement

Our research is based on public data from the NHANES; all details are from the official website (https://www.cdc.gov/nchs/nhanes/index.htm). To obtain the application executable files, please contact the author Zeru Chen by email: zrchan007@126.com.

## Author contributions

ZC: Conceptualization, Data curation, Formal analysis, Methodology, Software, Visualization, Writing – original draft, Writing – review & editing. ZW: Data curation, Investigation, Writing – original draft. YZ: Conceptualization, Methodology, Resources, Supervision, Funding acquisition, Writing – review & editing.
